# Segmentation-Assisted Fully Convolutional Neural Network Enhances Deep Learning Performance to Identify Proliferative Diabetic Retinopathy

**DOI:** 10.3390/jcm12010385

**Published:** 2023-01-03

**Authors:** Minhaj Alam, Emma J. Zhao, Carson K. Lam, Daniel L. Rubin

**Affiliations:** 1 School of Medicine, Stanford University, Stanford, CA 94305, USA; 2 Department of Electrical and Computer Engineering, University of North Carolina at Charlotte, Charlotte, NC 28223, USA

**Keywords:** AI in ophthalmology, segmentation aided classification, diabetic retinopathy, deep learning, computer aided diagnosis

## Abstract

With the progression of diabetic retinopathy (DR) from the non-proliferative (NPDR) to proliferative (PDR) stage, the possibility of vision impairment increases significantly. Therefore, it is clinically important to detect the progression to PDR stage for proper intervention. We propose a segmentation-assisted DR classification methodology, that builds on (and improves) current methods by using a fully convolutional network (FCN) to segment retinal neovascularizations (NV) in retinal images prior to image classification. This study utilizes the Kaggle EyePacs dataset, containing retinal photographs from patients with varying degrees of DR (mild, moderate, severe NPDR and PDR. Two graders annotated the NV (a board-certified ophthalmologist and a trained medical student). Segmentation was performed by training an FCN to locate neovascularization on 669 retinal fundus photographs labeled with PDR status according to NV presence. The trained segmentation model was used to locate probable NV in images from the classification dataset. Finally, a CNN was trained to classify the combined images and probability maps into categories of PDR. The mean accuracy of segmentation-assisted classification was 87.71% on the test set (SD = 7.71%). Segmentation-assisted classification of PDR achieved accuracy that was 7.74% better than classification alone. Our study shows that segmentation assistance improves identification of the most severe stage of diabetic retinopathy and has the potential to improve deep learning performance in other imaging problems with limited data availability.

## 1. Introduction

Diabetic retinopathy (DR) is the second leading cause of blindness in U.S. adults. In diabetic patients, retinopathy occurs when elevated blood glucose levels damage blood vessels in the eye, resulting in a variety of lesions that subsequently damage the retina. These lesions are readily visible in fundoscopy and color fundus photography. This includes microaneurysms, intraretinal hemorrhages, accumulation of lipid exudates due to capillary leakage, and neovascularization (NV; formation of new, abnormal blood vessels), among other lesions [1]. Severity of disease is based on the presence of different lesions, some of which can be detected by deep learning computer algorithms [2], and the number of eye regions affected. In this context, the identification of NV at the disc (NVD) or elsewhere (NVE) constitutes the key criterion for the initiation of panretinal photocoagulation [3], or anti-vascular endothelial growth factor (anti-VEGF) therapy [4].

State of the art deep learning methods such as convolutional neural networks (CNNs) can accurately classify disease status from imaging data for multiple conditions, such as in skin cancer, diabetic retinopathy, and breast cancer, to name a few [5,6,7]. CNNs are algorithms that learn from labeled training data. For imaging data this involves analyzing progressively more complex visual elements leading up to eventual categorization of whole images. Accuracy of these algorithms matches or exceeds those of human experts in recent studies. One such example is highlighted by Ehteshami et al., analysis of the 2016 CAMELYON16 competition for automated solutions in detecting breast cancer lymph node metastases, with CNNs sweeping the top 19 spots [7]. For diabetic retinopathy specifically, recent studies have demonstrated CNN-based methods for DR classification in all disease spectrum [6]. However, existing research on DR classification has not yet achieved the granularity of distinguishing proliferative DR (PDR) on retinal photography with high diagnostic accuracy, the most severe stage of DR, defined by the presence of NV, which requires urgent treatment to prevent retinal detachment and irreversible blindness.

Although deep learning continues to show immense promise for clinical imaging applications, current methods require large amounts of labeled training data. For best results, training datasets often consist of several thousand high-quality labeled images, which are costly to obtain and unavailable for rare diseases. Aside from increasing data availability, improving current methods to achieve similar results with fewer data requirements offers a possible solution to these constraints. A number of approaches can improve CNN results using fewer training data. The architecture of CNNs can be adjusted by adding different data processing layers (such as RELU and convolution layers). Images can also be pre-processed manually (e.g., by adjusting brightness or color) to bring out salient features before undergoing classification by the CNN. A separate deep learning algorithm, which historically has not been used in a pre-processing capacity, is the fully convolutional neural network (FCN) first described by Shelhamer et al. in 2017. While CNNs classify whole images into image categories, FCNs assign category labels to each pixel in an image. By labeling individual pixels, these networks perform image segmentation, separating clinically relevant portions of the image from other image regions.

Although FCNs have been successfully used to solve object localization problems [8,9,10], their potential as a pre-processing step for classification remains relatively unexplored—a paradigm we call “segmentation-assisted classification”. Especially, to the best of our knowledge, there is no established work that explores segmentation-assisted classification to identify DR progression to PDR. While traditional pre-processing methods rely on human identification of salient features, the deep-learning nature of FCNs could make them ideal candidates for processing images to identify the associated pathologies from the images prior to CNN classification.

Our study demonstrates the value of FCN segmentation-assisted classification of PDR, by segmenting NV specifically through deep learning, and successfully using FCN segmentation of NV in DR images for improved CNN classification of DR (non-proliferative NPDR vs. PDR).

## 2. Materials and Methods

### 2.1. Datasets and Image Selection

The Kaggle dataset (EyePacs, Santa Cruz, CA, USA), was used for developing and validating the model developed in this study. Kaggle contains retinal photographs (30°) from patients with varying degrees of severity of diabetic retinopathy and varying resolutions from 433 × 289 up to 5184 × 3456 pixels. The fundus photographs were colored, and it was made sure that the fovea and optic discs were visible in the images. Images from the dataset are already labeled with stage of disease, 1–4, following the diagnostic criteria for diabetic retinopathy. Stage 4, or proliferative diabetic retinopathy (PDR), is defined by the presence of neovascularization; patients at stage 3 and below have other lesions but lack neovascularization. These patients have non-proliferative diabetic retinopathy (NPDR).

Images from the dataset varied in quality, label accuracy, and image size. To address these discrepancies, images were discarded if they were poor quality (exclusion criteria: artifacts, image too dark or blurry to confidently stage disease, and if either fovea or optic disk was missing from the image). The exclusion criteria were set by an ophthalmologist.

1163 non-discarded images were reviewed, and NV annotations were corrected by a board-certified ophthalmologist if required. Stage 4 images were separated into those displaying active NV, and those without active NV who were nonetheless labeled stage 4 due to the presence of scars from laser treatment of past NV. Only those with active NV were labeled as such. Of the 1163 images, 60% of them were randomly assigned for use in segmentation. Out of that 60%, the segmentation model used 96% of the data for training and 4% of the data as training and validation set to evaluate segmentation performance. All photographs in the segmentation set were used to train the CNN classifier. These segmentation photographs were used for training only; none were used for classifier validation or testing. Finally, images were resized to a uniform pixel dimension of 512 × 512.

Research manuscripts reporting large datasets that are deposited in a publicly available database should specify where the data have been deposited and provide the relevant accession numbers. If the accession numbers have not yet been obtained at the time of submission, please state that they will be provided during review. They must be provided prior to publication.

Interventionary studies involving animals or humans, and other studies that require ethical approval, must list the authority that provided approval and the corresponding ethical approval code.

### 2.2. Labeling Neovascularizations

NV in diabetic retinopathy describes the formation of ectopic blood vessels outside the retina, distinguishable from normal healthy vessels by their thin and web-like appearance. NV technically differs from intraretinal microvascular abnormalities (IRMA), which are the dilation of existing vessels or formation of new ones within the retina and tend to appear more tortuous. IRMA marks stage 3, the most severe stage of NPDR, before the appearance of NV which marks stage 4, or PDR. Due to similar appearances the two lesions can be difficult to distinguish. IRMA may in fact be a precursor of NV, although this has not been proven [11]. For these reasons, severe IRMA was also included with NV during the process of mask generation described below.

**Figure 1:**
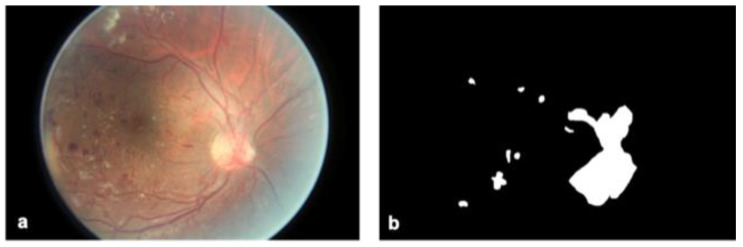
NV mask example. A retinal photograph (**a**) is shown with the NV mask created for it by the research team (**b**). White pixels in the mask (**b**) indicate areas of active NV or severe IRMA. Black pixels indicate areas without NV. This example shows NV clustering around the optic nerve.

For each image, a binary mask indicating the presence or absence of NV (or severe IRMA) was generated. To do this, the program GIMP was used to outline areas of NV over the original image and label all pixels within those areas with the number 1. All the annotations were conducted by a board-certified ophthalmologist and a trained medical student. The final decision on all the annotations was taken by the ophthalmologist. Pixels outside the areas of NV were labeled 0. Some images contained multiple distinct instances of NV while others contained none at all. Those without NV resulted in a mask labeled entirely with 0 s. Masks for 669 photographs were manually generated in this fashion (Figure 1). All 669 images were then divided into training (*n* = 644) and validation sets (*n* = 25) for segmentation by the FCN. The green channel of all images was isolated for segmentation, and red and blue image channels discarded. The green channel was chosen for yielding the most accurate segmentation results, which is consistent with the fact that NVs are red on imaging.

### 2.3. FCN Segmentation of Neovascularizations

**Figure 2:**
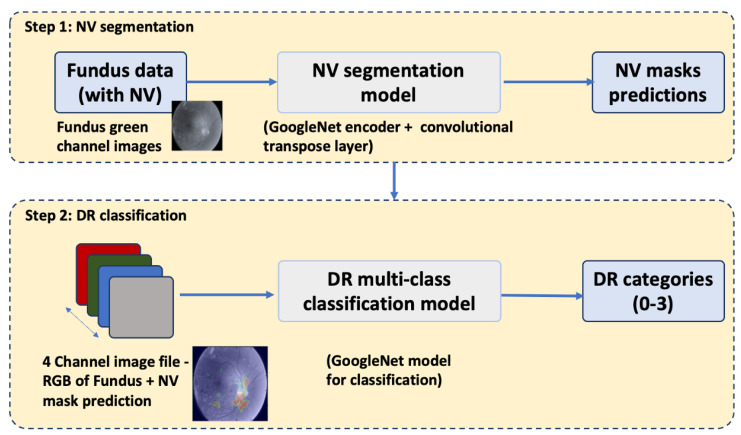
Steps of the segmentation-assisted DR classification algorithm. NV: neovascularization; DR: diabetic retinopathy.

A fully convolutional neural network (FCN) was constructed by replacing the last affine layer of the GoogleLeNet architecture with a convolutional transpose layer (deconvolution) that upsampled to the output dimensions of 512 × 512 × 2 using a stride of 32 and filter size of 64. The FCN was trained to segment retinal images for NV, i.e., label each individual pixel with a probability of representing neovascularization. Training and validation were performed on the 669 green-channel images described above, using the generated binary masks as correct labels. Training was implemented with the FirstAid Deep Learning repository implemented in Tensorflow 3.0 using Nvidia GeForce GTX 1080 ti graphical processing units (Nvidia, Santa Clara, CA, USA) (https://github.com/yidarvin/FirstAid, accessed on 11 March 2022). Training ran for 300 epochs, at a learning rate of 0.001, decay of 0.99, and dropout of 0.5. Accuracy was measured by calculating both the Dice score and the intersection over union (IOU) of true and predicted areas of segmentation. The segmentation model was saved to be used for classification in the next step. Overall steps of the segmentation-assisted algorithm is shown in Figure 2.

### 2.4. Baseline CNN Classification of Neovascularizations

In the classification step, a convolutional neural network (CNN) was trained to classify images into different categories of diabetic retinopathy with respect to NV. Four categories were outlined for this purpose: (1) NPDR which by definition lacks NV, (2) images with evidence of past (i.e., laser scars) but not active NV, (3) images with clinically ambiguous lesions that resembled NV (severe NPDR), and (4) confirmed NV (PDR). Of 1163 total images in the classification dataset, 75% were designated for training, 8% for validation, and 17% for testing (Table 1). Images with no NV made up the majority, followed by images with active NV.

Classification training, validation, and testing were performed on these 3-channel images. Similar to the FCN, the CNN used for classification was built with GoogLeNet architecture and trained with FirstAid Deep Learning implemented in Tensorflow 3.0 using Nvidia GeForce GTX 1080 ti graphical processing units. Data was augmented throughout the training and validation process by rotating, flipping, vertically and horizontally translating, and adjusting the brightness of images. Training ran for 300 epochs, at a learning rate of 0.001, decay of 0.99, and dropout of 0.5.

### 2.5. Segmentation-Assisted CNN Classification of Neovascularizations

Prior to classification, the previously trained and saved segmentation model was run on all green channel images in the training, validation, and testing sets. The resulting segmentation prediction, a 512 × 512 probability map with a probability of NV assigned to each pixel, was combined with the original 3 × 512 × 512 image to create a 4-channel image file. In this way segmentation information was added to the classification data without losing information contained in the original full-color (3-channel RGB) image. Classification training, validation, and testing were then performed on these 4-channel images following the same architecture, implementation, and hyperparameters as above. Segmentation prediction was run only once on classification images. All CNN classification trials used the same 4-channel images produced by this segmentation run. For each trial, the individual predicted labels and overall image classification accuracy were recorded.

### 2.6. Statistical Analysis

Differences in accuracy of the classification-assisted by segmentation approach compared to baseline (classification without segmentation) approach was assessed by averaging the accuracy of baseline and of segmentation-assisted trials. The accuracies were compared with a *t*-test comparison of means to determine statistical significance. For each category, sensitivity and specificity of the CNN classifier was computed across probability thresholds to plot receiver operating characteristic (ROC) curves and calculate area under the curve (AUC). Error analysis was calculated by computing standard deviation, shown in error bars.

### 2.7. Ethics

An institutional Review Board approval was not required for this study, which used data from a freely available public dataset containing no patient identifiable information.

## 3. Results

### 3.1. Segmentation of Neovascularization

**Figure 3:**
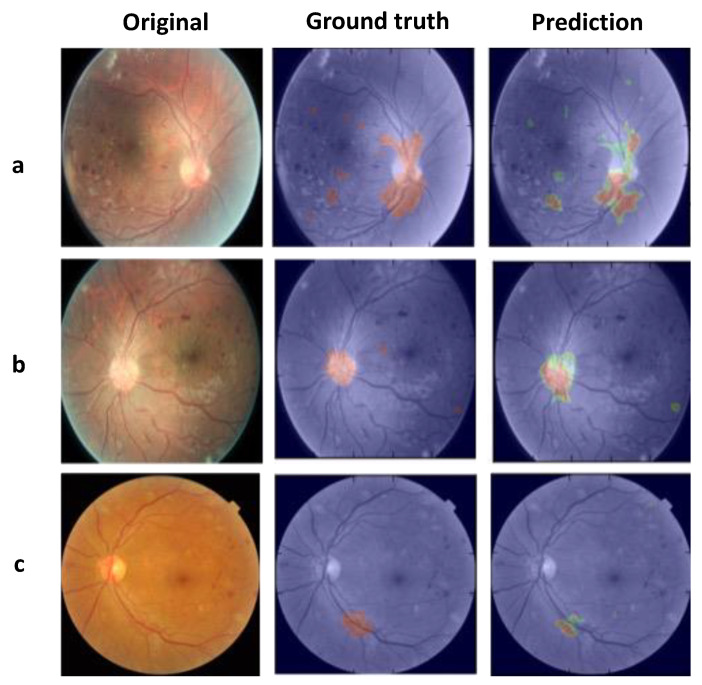
FCN-predicted NV compared to ground truth. ((**a**–**c**) are samples from three different patients with NV). In the Ground Truth panel, red shows areas of NV that were labeled on the NV mask by our research team, overlaid on the corresponding original retinal photograph. The Prediction panel depicts areas of NV predicted by the FCN after training on the photographs and NV masks. In the Prediction panel, red indicates a high probability that those pixels display NV, green indicates an intermediate probability, and blue a low probability.

For 669 total images in the segmentation validation set, the FCN detected NV regions of interest with a Dice score of 52.26% and intersection over union (IOU) of 42.26%. Many of these NV areas clustered around the optic nerve, which is visually distinct from the rest of the retina. The FCN model also detected areas of subtler NV, although with less confidence (not higher than 95%). Figure 3 depicts several retinal fundus photographs, their manually generated binary masks with true NV locations, and NV locations predicted by the segmentation network. The model performed excellent in identifying NV presence in an image with 95% accuracy.

### 3.2. Baseline and Segmentation-Assisted Classification

**Figure 4:**
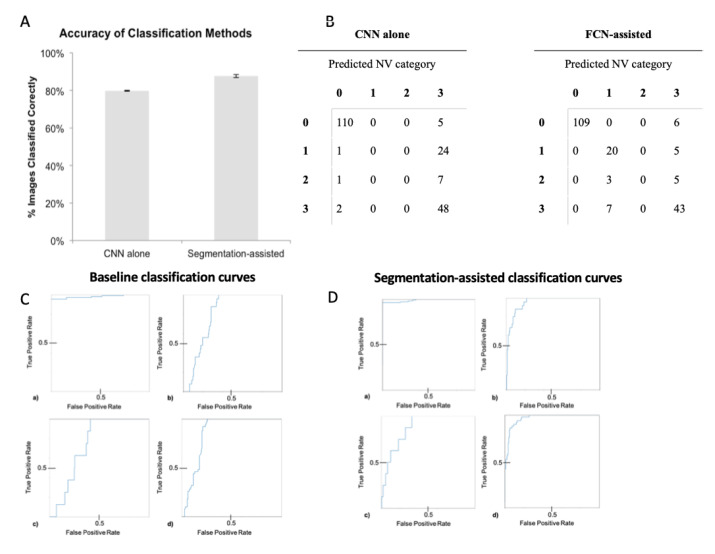
Performance analysis. (**A**) Average classification accuracy across three trials. The graph shows mean classification accuracy from three trials using CNN alone (79.97%, SD = 2.92%), compared to mean accuracy from three trials using segmentation-assisted classification (87.71%, SD = 7.71%). Error bars show ±SD; (**B**) Confusion matrix of image classification results; (**C**) Receiver operating characteristic (ROC) curves for classification by CNN alone. Four ROC curves are depicted; one for each classification category of DR: (a) Non-proliferative DR (area under the curve, AUC = 0.98), (b) Evidence of past NV (AUC = 0.8), (c) Ambiguous (AUC = 0.74), and (d) Proliferative DR with active NV (AUC = 0.86). (**D**) ROC curves for segmentation-assisted classification. Four ROC curves are depicted; one for each classification category of DR: (a) NPDR (AUC = 0.90), (b) Evidence of past NV (AUC = 0.95), (c) Ambiguous (AUC = 0.87), and (d) PDR with active NV (AUC = 0.97). The true positive rate is equivalent to sensitivity. The false positive rate is equivalent to 1—specificity.

For the baseline classification model, we used the CNN alone, without using the NV segmentation assistance. Baseline classification of images according to neovascularization was performed in three different trials (Figure 4A). The accuracy of baseline classification by CNN alone was 79.97% (SD = 2.92%). Figure 4B shows data from one trial of how the CNN classified each individual image in the test set, as compared with the ground truth (manual annotation). Baseline classification largely classified images into categories 0 (NPDR) or 3 (active NV). In our case, no images were classified into category 1 (inactive PDR following photocoagulation), and few into category 2 (vascularization resembling NV). Of note, images in category 0 generally had fewer lesions than those in category 3. Category 3 images were also more likely to have large non-NV lesions as well.

Segmentation-assisted classification incorporated prediction maps from the FCN segmentation process. Segmentation-assisted classification was run in 3 different trials, all using 512 × 512 prediction files produced by the same segmentation run prior to classification (Figure 4A). Figure 4B shows the confusion matrix for classification results in the test set, compared with the ground truth. The mean accuracy across trials of segmentation-assisted classification was 87.71% on the test set (SD = 7.71%). This showed a 7.74% improvement over classification by CNN learning alone, which was statistically significant (*p* = 0.0024). All three segmentation-assisted trials outperformed CNN-alone trials. Figure 4C,D demonstrate the receiver operation curves (ROC) for baseline and segmentation-assisted classification models in identifying classification categories 0 through 4. The area under the ROC curves (AUC) values are shown in Table 2.

The greatest improvement with segmentation assistance was in distinguishing images with past NV (category 1) from those with active NV (category 3). Compared to baseline, the segmentation-assisted classification approach labeled the majority of past NV images correctly (20 of 25 images, compared to 0 of 25). Of the few active NV images that were incorrectly classified, all were labeled as having past NV. The presence of active NV and scars from past treatment are not mutually exclusive; several patients with treatment scars in our dataset also had active NV. Of eight images with clinically ambiguous lesions (category 2), the segmentation-assisted classifier incorrectly labeled three as having active NV, and five as having past NV. Thus, the improvement in classification accuracy with segmentation assistance is due to both improved sensitivity and specificity across all NV categories (Figure 4).

## 4. Discussion

Neovascularization is an important marker for proliferative DR, which if left untreated, can result in vitreous hemorrhage, retinal detachment, and is strongly associated with neovascular glaucoma and thus requires urgent treatment to prevent irreversible vision loss [12]. In clinical management of DR, it is important to track the transition from severe NPDR to PDR for therapeutic intervention, i.e., use of anti-VEGF (vascular endothelial growth factor) treatment in the retina. In contrast to previous literature on deep learning-based ophthalmic diagnostics, our study combines the power of deep learning and NV-specific detection to distinguish proliferative DR with greater accuracy. Therefore, the novel utility of our algorithm lies in identifying the transition from NPDR to PDR on retinal photography, helping to facilitate early intervention to prevent vision impairment.

This study is the first to our knowledge to show a statistically significant improvement in classification accuracy (7.74%, Figure 4) on retinal photography when incorporating automated image segmentation produced by a trained FCN. Our results show that this improvement comes from increased sensitivity and specificity in detecting NV, therefore PDR images. The CNN classifier alone essentially learned to distinguish only category 0 images, likely by identifying obvious non-specific abnormalities (Figure 4B). Examples of obvious abnormalities include white fibrous tissue that often forms with NV and obscures underlying retina, and large hemorrhages that are more likely with severe disease. In contrast, after FCN segmentation of the images, the classifier was able to distinguish retinas with active NV (category 1) from those that received prior treatment but had no active lesions (category 3; Table 2). Thus, this ability to identify NV specifically most likely accounted for the 7.74% improvement in classification accuracy.

With diabetes incidence increasing worldwide, vision loss with diabetic retinopathy, especially PDR will be a rising global health problem [13,14]. Neovascularizations can be easily confused with other lesions such as IRMA, and missed if small. Yet, NV is crucial to treat before vision is permanently lost. In this context, there is an opportunity for deep learning diagnostic assistance to supplement clinician review of retinal photographs by highlighting images of particular concern. Moreover, it was recently established (in the setting of histopathologic classification of liver cancer) that deep learning may indeed improve the accuracy of clinicians when integrated in a clinical workflow [15].

The phenomenon of improved overall classification after first training on smaller specific features (i.e., NV) can be explained by the structure of CNNs and FCNs. Neural networks learn by processing multiple smaller visual motifs (e.g., lines, then corners, then squares) that together form an image [16]. To this end, FCNs are built on the same architecture as CNNs. The major difference, as first described in 2017 by Shelhamer et al., is the addition of an extra layer that classifies every individual pixel of an image by assigning it a category label [17]. An FCN can therefore be thought of as having trained on 512^2^ = 262,144 data points for a single 512 × 512 image. This provides many more training opportunities than direct CNN classification, in which every image can only serve as exactly one training point because labels are assigned to whole images. This explains why the learning of even a few small areas of NV by an FCN improved CNN learning in whole-image classification.

Similar to transfer learning, segmentation-assisted classification has the potential to address data limitations. Recent studies that show CNNs rivaling physicians in diagnostic accuracy used hundreds of thousands of images for training [5,6,7]. Segmentation increases the number of training opportunities without expanding the classification dataset by nature of the individual pixel-level training in segmentation and by the augmented classification abilities achieved when incorporating this information into classification tasks. Therefore, segmentation-assisted classification could be advantageous by providing the ability to increase diagnostic accuracy in cases where there is limited training data for use outside of common conditions like diabetic retinopathy as well. In rarer diseases with few available cases to learn from, less data-intensive techniques such as we describe provide the possibility of applying deep learning where it was previously impractical.

Recent trends in ophthalmic diagnostics have moved toward making teleophthalmology globally available and training datasets more demographically inclusive [14,18,19,20,21]. By decreasing focus on the overall image appearance, segmentation-assisted classification may also reduce classification bias when algorithms trained in one population are applied to another.

It is important to note that segmentation accuracy, as well as degree of classification improvement, will likely vary widely depending on the type of lesion. For example, microaneurysms are more discrete in nature and therefore easier to classify than neovascularizations; it thus follows that segmentation-assisted classification for microaneurysms may result in greater accuracy improvement. Research on segmentation-assisted classification with other lesions, especially those that are more easily demarcated than neovascularizations, may yield even better results.

The concept of applying information from one deep learning task to another has yielded favorable results in medical diagnostics. In transfer learning, as surveyed by Weiss et al. in 2016, an algorithm is trained on one task prior to training on a separate similar task, with the hypothesis that having prior related training will result in better learning [22]. Because of this ability to train on different tasks, transfer learning is a useful technique in low-data settings. It has been applied to problems of image detection, survival prediction, audio analysis, and prediction of molecular interactions, among others [23,24,25,26,27]. However, little published research uses transfer learning to apply image segmentation to medical classification problems. Additionally, while our study does use training on a previous task (segmentation) to optimize classification, it differs from transfer learning in the method of information incorporation. This study uses final predictions produced by the fully preserved FCN to inform classification, rather than subjecting the original neural network to complete retraining for a new task as in transfer learning. The classification improvement we observe with segmentation-assisted classification is most likely due to additional information in the form of the NV probability maps overlayed onto original images. Importantly, this suggests that for certain imaging problems, segmentation can assist the most currently refined CNNs to improve classification even from a high baseline accuracy. Analogously, Yim and coworkers used recently for the prediction of progression of age-related macular degeneration the combination raw optical coherence tomography imaging data and the output of a segmentation model as input for two prediction models, that were then ensembled.

There are some limitations of this study. As is the case for all supervised deep learning, FCNs and CNNs can only do as well as the accuracy of the labels provided. The gold standard for NV detection uses fluorescein angiography, a method of improving blood vessel visualization that was unavailable for our study. Despite the best effort of an expert clinician, every label in our dataset may not be perfect. If training images are erroneously labeled by human experts, algorithm performance will be impaired. One potential solution to this is to use extra-image data in determining labels. For example, if a patient with a clinically ambiguous lesion resembling NV presents at a follow-up visit with evident NV evolved from the earlier lesion, labeling photographs from the first visit can teach CNNs to detect early-stage NV below even an expert physician’s threshold for definitive detection. Information from electronic health records (e.g., blood sugar readings) can also inform labeling of otherwise ambiguous lesions.

Our model is also only applicable to photography data and can be limited in terms of utilizing other patient metadata such as electronic health report (EHR) data. This can be a future direction where image data can be integrated with HER data. Other limitations of our study include the labor-intensive need for clinical experts to generate a substantial number of detailed segmentations. The subjectivity of this process varies greatly by lesion. Neovascularizations, for instance, have much less discrete boundaries than do microaneurysms, which are well-circumscribed lesions that appear early in diabetic retinopathy. Even with high-quality segmentations, it is difficult to ensure that classification prioritizes segmented lesions over potentially distracting image components. For example, scars from past NV treatment are much more visually evident than NV, but it is the presence of active NV that defines active proliferative DR—a fact our results show can be confusing to classifiers.

Unlike transfer learning, the use of segmentation to assist a classification task raises the question of how to best incorporate this information into deep learning models. In addition to adding the segmentation as a fourth image channel, other possibilities include brightening or increasing contrast in segmented areas. One other alternative can be using segmentation as a self-supervised task, that teaches the deep learning model subtle embeddings to classify different disease stages in a subsequent downstream task. Further work comparing segmentation-assisted classification to transfer learning and self-supervised learning will yield insight into the merits of each technique. Furthermore, comparison of CNN-alone and segmentation-assisted classification to physician accuracy on a larger set of images will inform the value of using such algorithms in clinical ophthalmology practice. There are other areas for research to explore the use of segmentation-assisted classification, such as combining the results of segmentations for multiple lesion types (e.g., for microaneurysms, hemorrhages, and exudates), and comparing results to transfer learning methods with the potential to combine components of each.

Ultimately, based on our promising results, segmentation-assisted classification holds promise for applications beyond retinal NV to other imaging modalities and diseases. With the growing field of research on neural networks in medical imaging, there are rich opportunities to explore these applications.

## 5. Conclusions

We present a deep learning-based segmentation-assisted classification model to identify NV in PDR, which is the most clinically severe stage of DR. We found that incorporating results from automated image segmentation improved classification accuracy of proliferative DR images. This technique of segmentation-assisted classification holds promise for other imaging problems including those with limited available training data. Beyond imaging, the use of multiple algorithms to complement each other warrants continued research for its potential to improve all forms of deep learning problems.

## Figures and Tables

**Table 1:** Image categories. Most images (52%) had no NV. 32% had active NV, while 12% had evidence of treatment for previous NV but no active lesions; a minority (4%) had clinically ambiguous lesions.

	Training	Validation	Testing	Total for Category (%)
0: NPDR, no NV	434	55	115	604 (52%)
1: Past NV	103	13	25	141 (12%)
2: Ambiguous	31	3	8	42 (4%)
3: NV	299	27	50	376 (32%)
Total for task	867	98	198	1163

**Table 2:** Area under the ROC curves (AUC) values for DR classification tasks.

	Baseline	Segmentation Assisted
0: NPDR, no NV	0.98	0.9
1: Past NV	0.8	0.95
2: Ambiguous	0.74	0.87
3: NV	0.86	0.97

## Data Availability

The datasets generated during and/or analyzed during the current study are available in the Kaggle repository, https://www.kaggle.com/c/diabetic-retinopathy-detection/data (accessed on 25 October 2022).
